# Establishing a patient-derived xenograft model of human myxoid and round-cell liposarcoma

**DOI:** 10.18632/oncotarget.17352

**Published:** 2017-04-21

**Authors:** Yiming Qi, Yu Hu, Hua Yang, Rongyuan Zhuang, Yingyong Hou, Hanxing Tong, Yi Feng, Yuan Huang, Quan Jiang, Qunsheng Ji, Qingyang Gu, Zhixiang Zhang, Xuzhen Tang, Weiqi Lu, Yuhong Zhou

**Affiliations:** ^1^ Departments of Geriatrics, Zhongshan Hospital, Fudan University, Shanghai, China; ^2^ Departments of General Surgery, Zhongshan Hospital, Fudan University, Shanghai, China; ^3^ Departments of Oncology, Zhongshan Hospital, Fudan University, Shanghai, China; ^4^ Departments of Pathology, Zhongshan Hospital, Fudan University, Shanghai, China; ^5^ Endoscopy Center, Zhongshan Hospital, Fudan University, Shanghai, China; ^6^ Oncology BU, Research Service Division, WuXi AppTec, Shanghai, China

**Keywords:** liposarcoma, MRCL, PDX model, PIK3CA mutation, PI3K/mTOR pathway

## Abstract

Myxoid and round cell liposarcoma (MRCL) is a common type of soft tissue sarcoma. The lack of patient-derived tumor xenograft models that are highly consistent with human tumors has limited the drug experiments for this disease. Hence, we aimed to develop and validate a patient-derived tumor xenograft model of MRCL. A tumor sample from a patient with MRCL was implanted subcutaneously in an immunodeficient mouse shortly after resection to establish a patient-derived tumor xenograft model. After the tumor grew, it was resected and divided into several pieces for re-implantation and tumor passage. After passage 1, 3, and 5 (i.e. P1, P3, and P5, respectively), tumor morphology and the presence of the FUS-DDIT3 gene fusion were consistent with those of the original patient tumor. Short tandem repeat analysis demonstrated consistency from P1 to P5. Whole exome sequencing also showed that P5 tumors harbored many of the same gene mutations present in the original patient tumor, one of which was a *PIK3CA* mutation. PF-04691502 significantly inhibited tumor growth in P5 models (tumor volumes of 492.62 ± 652.80 vs 3303.81 ± 1480.79 mm^3^, *P* < 0.001, in treated vs control tumors, respectively) after 29 days of treatment. In conclusion, we have successfully established the first patient-derived xenograft model of MRCL. In addition to surgery, PI3K/mTOR inhibitors could potentially be used for the treatment of *PIK3CA*-positive MRCLs.

## INTRODUCTION

Liposarcoma is one of the most common soft tissue sarcomas. It can be classified into four biological groups [1, 2]: well-differentiated liposarcoma, myxoid and round-cell liposarcoma (MRCL), pleomorphic liposarcoma, and de-differentiated liposarcoma. Among them, MRCLs are predominant accounting for nearly 33% of all cases [3]. In general, morphologically round-cell liposarcomas are more aggressive than pure myxoid tumors, and a high percentage of round cells is often correlated with poor prognosis. MRCLs are characterized by unique chromosomal translocations and fusions, commonly harboring the t(12;16) (*FUS-DDIT3*) fusion; however, the t(12;22) (*EWSR1-DDIT3*) fusion can also be present. These gene fusions exist only in MRCLs, indicating a high specificity for diagnosis. Clinically, primary MRCL tumors usually arise in the limbs, and rarely from retroperitoneal or subcutaneous tissues like other types of liposarcomas. These tumors commonly metastasize to serosal membranes (peritoneum, pleura and pericardium, and abdominal cavity), distant soft tissues, and bone [4]. Surgical resection remains the most common treatment for MRCL. However, local recurrence and early distant metastasis significantly reduce patient quality of life and long-term survival, and traditional chemo- and radiation-based therapies show limited therapeutic effects.

PI3K is activated by tyrosine kinase growth factor receptors; this leads to phosphorylation of downstream mediators such as Akt and mTOR. Those two factors then promote phosphorylation of targets such as S6 ribosomal protein [5]. The abnormal activation of PI3K/Akt is thought to be an essential step in the initiation and survival of many human tumors. Inhibitors of PI3K signaling have proven to be beneficial for the treatment of many malignancies including breast cancer and non-small cell lung cancer, among others [6, 7]. *PIK3CA* encodes the catalytic subunit of phosphatidylinositol 3-kinase (PI3K), and mutations often activate PI3K signaling. Such mutations have been reported to occur in 18% of MRCLs [8], and are associated with round cell transformation and poor clinical outcomes [9].

To date, the biological behavior and pathophysiology of MRCL are poorly understood, and as such, pre-clinical drug trials for this disease are rare. One of the major limitations is the lack of animal models that are comparable to human MRCL. To our knowledge, three MRCL animal models have been reported. All of these models were established using a mesenchymal stem cell line or a MRCL cell line harboring the FUS-DDIT3 gene fusion [10–12]. It is well established that the FUS-DDIT3 chimeric protein is associated with human liposarcoma. These models were valuable for studies on the *in vivo* function of FUS-DDIT3. However, they did not fully recapitulate the features of human liposarcoma or the cell-cell interactions that occur in human tumors; this limited their value for studies on targeted drug treatments.

Patient-derived xenograft (PDX) models have shown utility in recent years. For these, a tumor mass from a patient is directly implanted into the subcutaneous tissue of a severe combined immunodeficiency (SCID) mouse, which is followed by tumor growth. Upon reaching a sufficient mass, tumors are explanted, divided into smaller samples, and re-implanted for subsequent passage in mice. Therefore, a small piece of tumor can be propagated to achieve a tumor mass many-fold greater than that of the original tumor. Using this principle, the original tumor mass can be reproduced after many passages. Comprehensive genome-wide gene expression analyses have shown that tumors from PDX models at an early passage have genomic expression profiles that are very close to those of the original tumors [13]. Using PDX models, physicians have selected specific therapeutic agents to utilize for patient tumors. Theoretically, this approach could thus be used for personalized patient treatment, especially when the traditional standard of care fails. Researchers have tried to identify key pathway components that are suitable for targeted drug development [14]. However, the establishment of a PDX model is very expensive, requires a high level of technical support, and is associated with a low success rate, thus limiting its application. To our knowledge, no MRCL PDX model has been successfully established.

In the current study, we attempted to establish a PDX model of MRCL. Key features of the model were validated after passaging by assessing histological morphology and by confirming the presence of the FUS-DDIT3 gene fusion. Serially passaged tumors were compared to the original tumor sample from the patient. In addition, our patient tumor sample was positive for a *PIK3CA* mutation. We therefore utilized the PI3K inhibitor PF-04691502 to further validate our PDX model of MRCL.

## RESULTS

### Patient information

The donor sample used to establish the PDX model came from a 47-year-old man. The tumor was first found on the right thigh of the patient in 2006; after complete resection, it returned in 2009 both in the original location and in the retroperitoneum. The tumor was excised again, but reappeared twice in the retroperitoneum until complete surgical resection was impossible in 2013. Before that, no chemo or radiation therapy was administered. Histological analysis of the tumor samples showed that they were consistent with MRCL. The sample used to establish the PDX model in the current study was obtained from the second-to-last operation in July 2012.

### PDX model of MRCL

**Figure 1:**
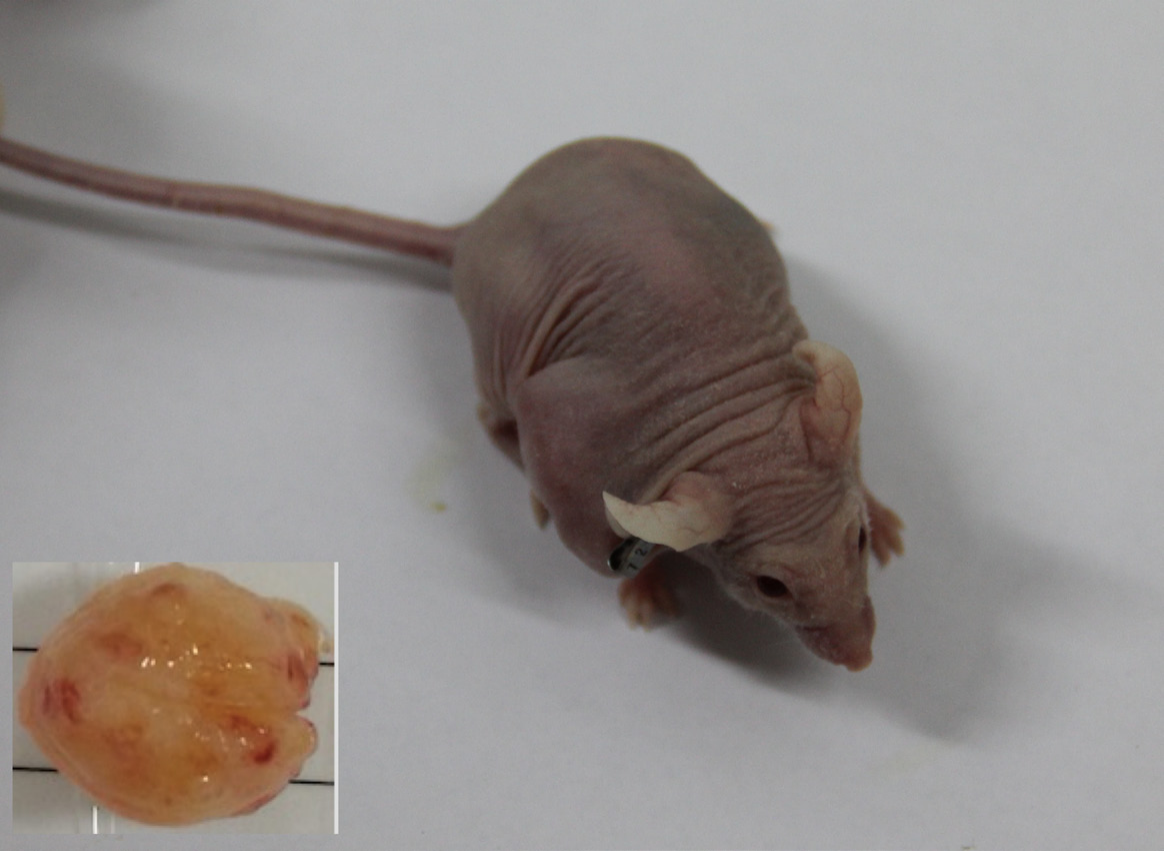
Depiction of myxoid and round cell liposarcoma patient-derived xenograft model and the gross specimen of the tumor

The primary MRCL tumor (P0) was propagated for five passages (P1–P5). We randomly examined xenograft mice from each passage. Based on gross morphology, all xenografts showed preserved features of the original tumor (P0) (Figure 1).

Tumor histology and the presence of the DDIT3 gene were examined using samples from P1, P3, and P5. These samples were compared to the patient tumor sample.

**Figure 2:**
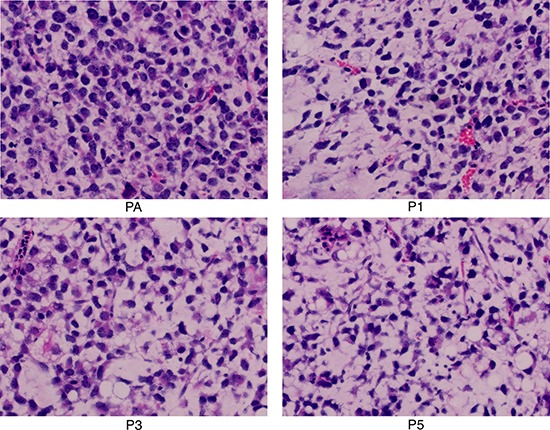
Representative hematoxylin and eosin sections of patient tumor sample (PA) and myxoid and round cell liposarcoma patient-derived xenograft tumor samples (P1, P3, P5) (20×)

Hematoxylin and eosin (H&E) staining showed that P1, P3, and P5 tumor samples were round cell liposarcoma with signet ring cells in the mucous background. These characteristics are specific for MRCL, as shown in the patient tumor sample (Figure 2).

**Figure 3:**
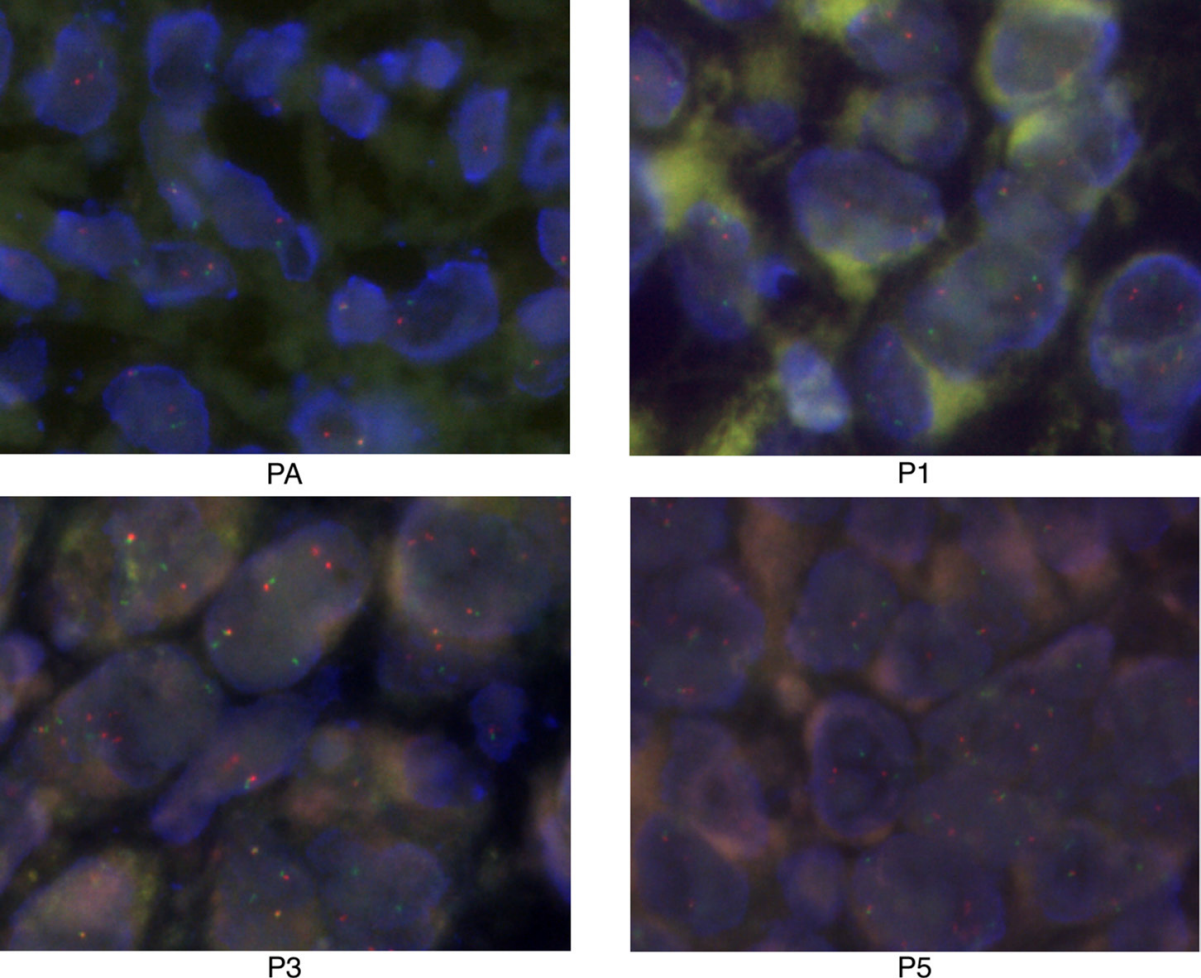
Fluorescence *in situ* hybridization results of the patient tumor sample (PA) and the patient-derived xenograft tumor samples (P1, P3, P5) (40×) The red and green point was labeled on both ends of the *DDIT3* gene; the distance between the two colors indicates a translocation of this gene.

Fluorescence *in situ* hybridization (FISH), specific for the FUS-DDIT3 gene fusion, showed that P1, P3, and P5 tumor samples had similar positivity to the patient sample (Figure 3).

**Figure 4:**
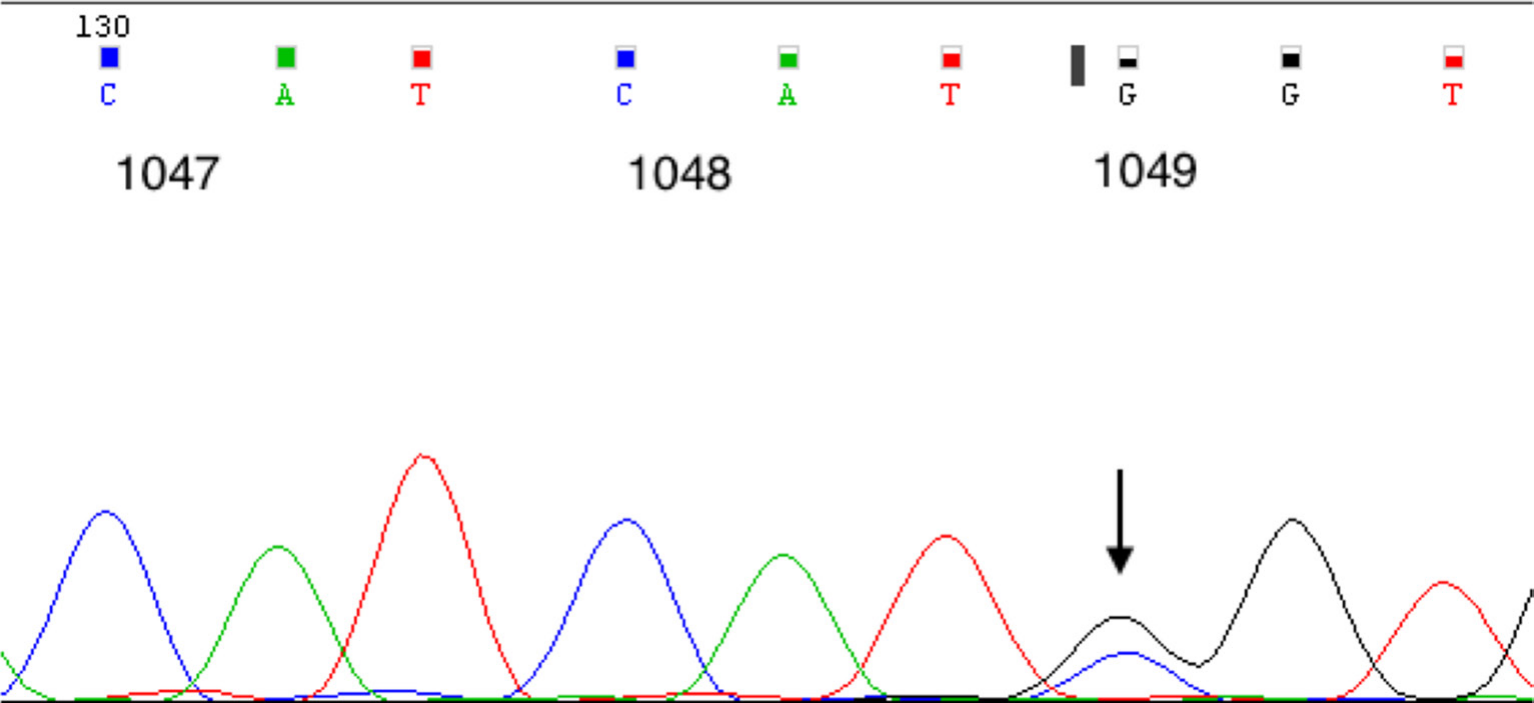
*PIK3CA* genotyping of the patient tumor sample that was used to establish the myxoid and round cell liposarcoma patient-derived xenograft model A mutation was identified at exon 20; cDNA: G3145C, protein: G1049R.

When comparing tumor-specific gene mutations between P5 and the original patient tumor (P0) samples using whole exome sequencing (WES), we found that the two were nearly identical (Table 1). According to WES results, a *PIK3CA* mutation was detected in both the P5 model tumor and the original patient tumor, Sanger sequencing was used to confirm the *PIK3CA* mutation in the original patient tumor (Figure 4).

**Table 1:** Shared mutations of myxoid and round cell liposarcoma (MRCL) patient-derived xenograft (PDX) model and the original patient tumor sample

Gene Symbol	Original patient tumor sample	Corresponding PDX model sample
PIK3CA	Exon 20: cDNA: G3145C, protein: G1049R	Exon 20: cDNA: G3145C, protein: G1049R
TET2	Exon 3: cDNA: T320A, protein: L107H	Exon 3: cDNA: T320A, protein: L107H
	Exon 7: cDNA: C3809T, protein: T1270I	Exon 7: cDNA: C3809T, protein: T1270I
KAT6A	Exon 18: cDNA: G3937A, protein: D1313N	Exon 18: cDNA: G3937A, protein: D1313N
	Exon 17: cDNA: G3937A, protein: D1313N	Exon 17: cDNA: G3937A, protein: D1313N
FGFR2	Exon 6: cDNA: A877G, protein: K293E	Exon 6: cDNA: A877G, protein: K293E
	Exon 6: cDNA: A868G, protein: K290E	Exon 6: cDNA: A868G, protein: K290E
	Exon 7: cDNA: A868G, protein: K290E	Exon 7: cDNA: A868G, protein: K290E
	Exon 7: cDNA: A946G, protein: K316E	Exon 7: cDNA: A946G, protein: K316E
KDM5A	Exon 21: cDNA: C3091T, protein: R1031C	Exon 21: cDNA: C3091T, protein: R1031C
BRIP1	Exon 5: cDNA: G430A, protein: A144T	Exon 5: cDNA: G430A, protein: A144T

Note: The table exhibits the alteration and location of the mutations detected by whole exome sequencing (WES). For example: Exon 20: cDNA: G3145C, protein: G1049R means the 3145th base sequence in exon 20 has mutated from G to C, and the corresponding protein's 1049th amino acid has mutated from glycine to arginine. Due to limited space, we have only listed some tumor related, nonsynonymous mutations on the table.

Short tandem repeat (STR) analysis of P1–P5 specimens showed that after detecting repetitive sequences in 10 STR sites, the similarity between P1 and P3 was 100%, P3 and P4 was 80%, and P4 and P5 was 100% (Table 2).

**Table 2:** Short tandem repeat (STR)-analysis of P1–P5 at 10 genetic sites

Genetic Site	P1	P2	P3	P4	P5
Amelogenin	X, Y	X, Y	X, Y	X, Y	X, Y
CSF1PO	9, 11	9, 11	9, 11	11, 13	11, 13
D13S317	8, 8	8, 8	8, 8	8, 11	8, 11
D16S539	11, 12	11, 12	11, 12	9, 12	9, 12
D5S818	13, 13	13, 13	13, 13	13, 13	13, 13
D7S820	11, 12	11, 12	11, 12	8, 11	8, 11
THO1	7, 7	7, 7	7, 7	7, 7	7, 7
TPOX	8, 10	8, 10	8, 10	8, 10	8, 10
vWA	17, 19	17, 19	17, 19	17, 19	17, 19
D21S11	30, 32.2	30, 32.2	30, 32.2	30, 32.2	30, 30.2

Note: The number of the samples shown for each genetic site is the repeat count of the repetitive sequence.

### PF-04691502 Treatment

**Figure 5:**
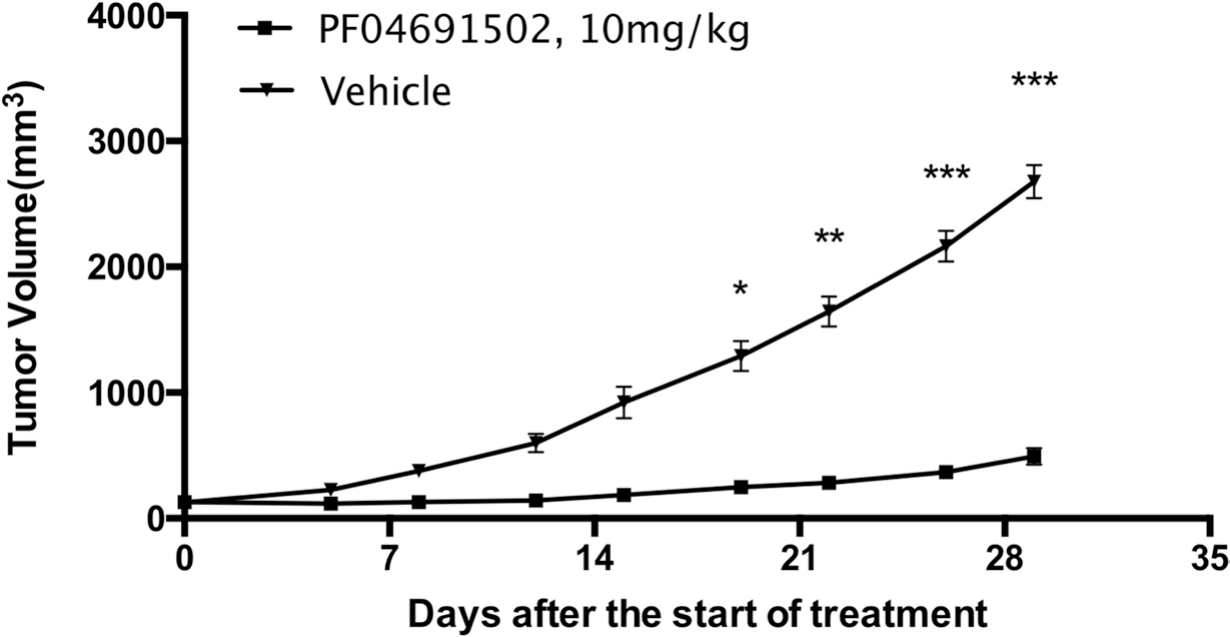
*In vivo* efficacy of PF-04691502 in myxoid and round cell liposarcoma patient-derived xenograft model (*n* = 10) This compound exhibited profound anti-tumor activity compared to that observed for the vehicle group (*n* = 10) after administration for 19 days and longer (data are represented as mean ± SEM, *P < 0.05, **P < 0.01, ***P < 0.001).

We used the PI3K/mTOR inhibitor PF-04691502 to test our MRCL PDX model. The trial began when the average tumor volume in the PF-04691502 treatment group was 130.00 ± 29.28 mm^3^ and that in the vehicle-only control group was 128.25 ± 32.78 mm^3^. After a 29-day treatment, the average tumor size in the PF-04691502 group was 492.62 ± 652.80 mm^3^, whereas that in the control group was 3303.81 ± 1480.79 mm^3^. Thus, the tumors in the treatment group were significantly smaller than those in the control group (*P* < 0.001) (Figure 5).

**Figure 6:**
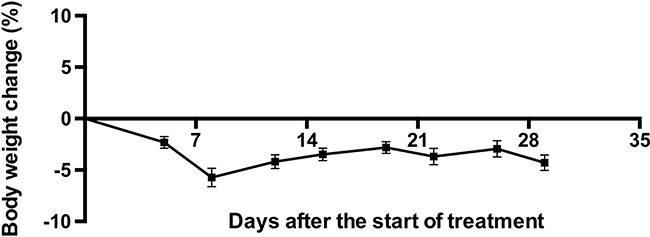
The influence of PF-04691502 on mouse body weight, which could indicate side effects of the drug (*n* = 10, data are represented as mean ± SEM)

Mice were found to tolerate PF-04691502 very well. Average weight loss in the treatment group mice was below 10% (Figure 6).

**Figure 7:**
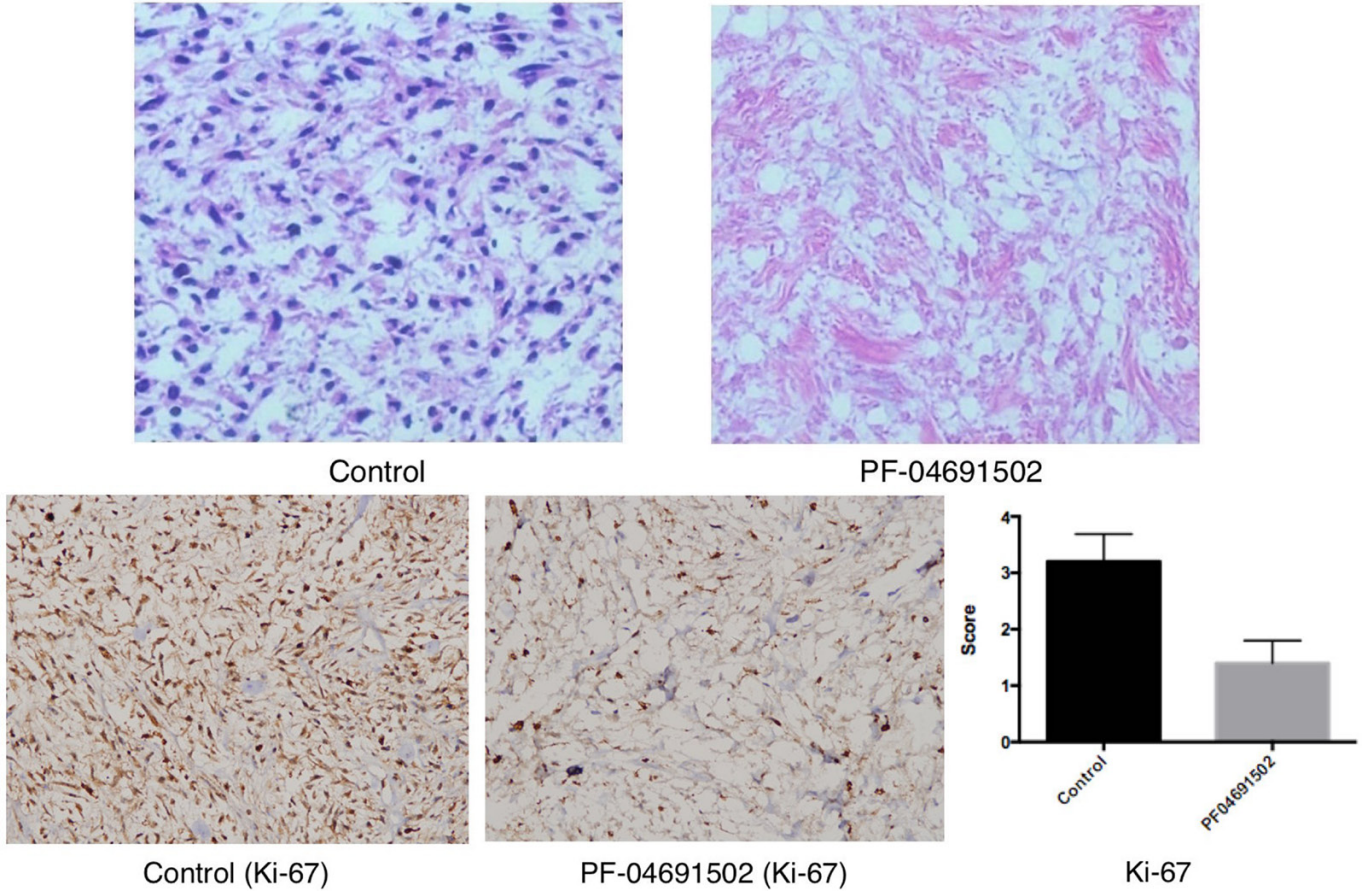
Hematoxylin and eosin (20×) and Ki-67 (10×) staining of tumors from the vehicle and PF04691502 groups from the myxoid and round cell liposarcoma patient-derived xenograft model The PF04691502 group showed a large amount of acellular tissue and lower expression of Ki-67 compared to those in the control group (*P* = 0.022).

Furthermore, H&E staining showed that PF-04691502 treatment resulted in significantly fewer viable cells compared to that with vehicle treatment, and Ki-67 positivity was significantly lower in the PF-04691502 group than in the control group (1.40 ± 0.89 vs 3.20 ± 1.10, respectively, *P* = 0.022) (Figure 7).

**Figure 8:**
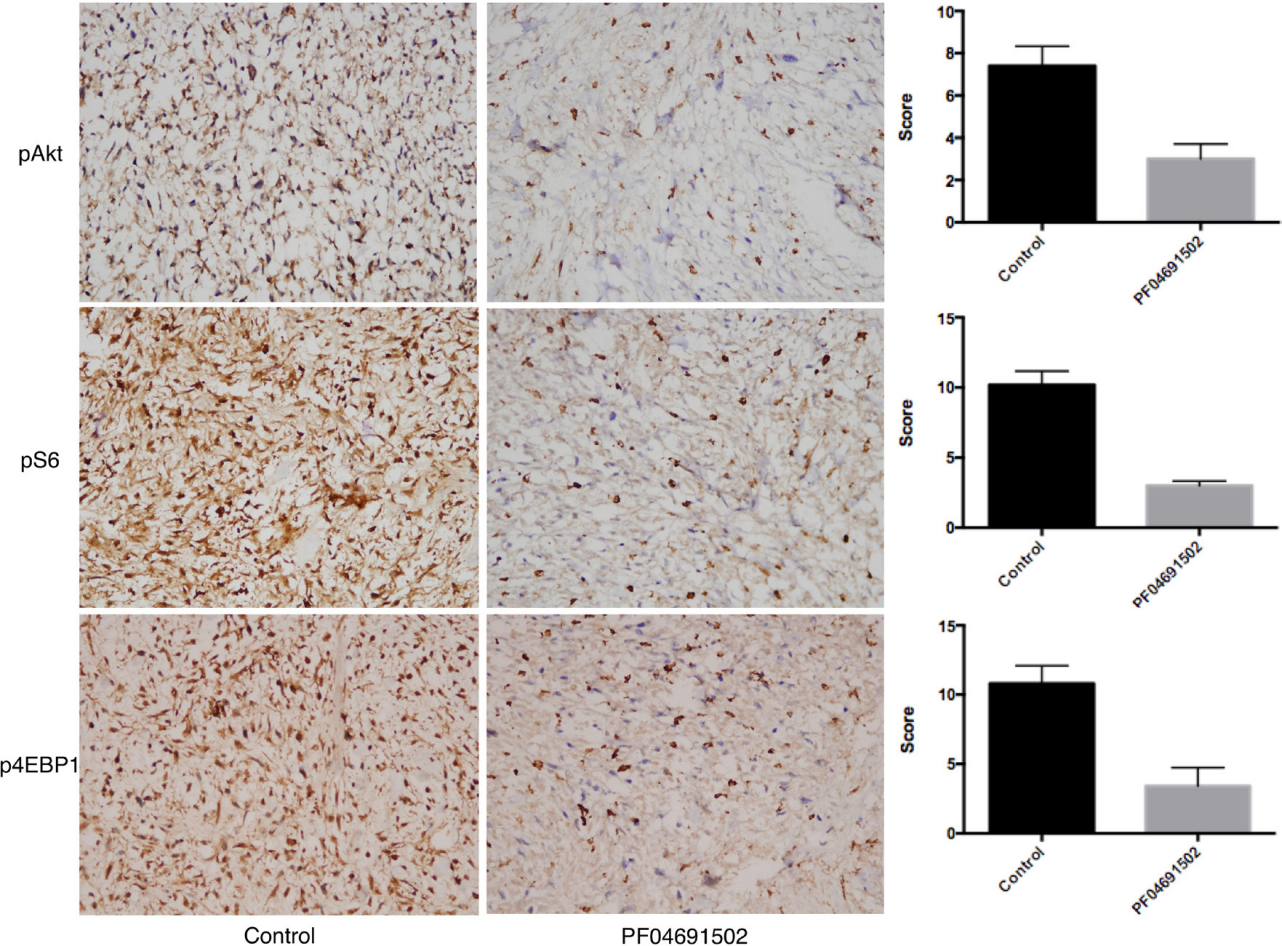
Immunohistochemical staining for pAkt, pS6, and p4EBP1 in tumors from the vehicle and PF04691502 groups of the myxoid and round cell liposarcoma patient-derived xenograft model The results show that the expression of all three markers was significantly lower in the PF04691502 group than in the vehicle group (*P* = 0.005, *P* = 0.001, *P* = 0.001) (10 × magnification for images shown).

Immunohistochemistry showed that the expression of pAkt, pS6, and p4EBP1 was significantly lower in the PF-04691502 group than in the control group (pAkt: 3.00 ± 1.58 vs 7.40 ± 2.07, respectively, *P* = 0.005; pS6: 3.00 ± 0.71 vs 10.20 ± 2.17, respectively, *P* = 0.001; p4EBP1: 3.40 ± 1.34 vs 10.80 ± 2.86, respectively, *P* = 0.001) (Figure 8).

Western blotting showed no difference in the expression of PPARgamma and no band was observed for ap2 or adipsin in either the PF-04691502 or control group (data not shown).

## DISCUSSION

Palliative chemotherapy, including doxorubicin combined with ifosfamide, is usually required for MRCL patients [15]. However, when this fails, there is no effective secondary treatment. Furthermore, chemotherapy is often associated with considerable toxicity. Thus, new and effective targeted therapies are desperately needed for MRCL treatment. However, the lack of a proper animal model has hampered advances in creating and testing such therapeutics.

In our study, we successfully established a PDX model of human MRCL. We validated our model by histological analysis, FISH, WES assays, and STR analysis. We demonstrated that our model tumors have identical features to the original patient tumor, and these features are preserved until at least P5. Our PDX model of human MRCL is believed to be the first of its kind.

WES results showed that our patient tumor sample had a *PIK3CA* mutation, and this was also true of the PDX model. Jordi et al. [8] found that 18% of MRCLs harbor *PIK3CA* mutations, which are believed to be responsible hyperactivation of PI3K/mTOR signaling. Guo and colleagues [16] also found that 22% of liposarcomas have *PIK3CA* mutations, and these all proved to be MRCLs. Demicco et al. [9] showed that activation of the PI3K pathway might induce round cell transformation of MRCLs. We demonstrated that tumor samples from the donor patient and those from the mouse model (P5 as representative) were both positive for a *PIK3CA* mutation. This finding not only validated our PDX model, but also indicates that this animal model is suitable for testing potential treatments for liposarcoma.

We chose the PI3K/mTOR dual inhibitor PF-04691502 to test using our PDX model. PI3K/mTOR pathway plays a key role in regulating cell growth, cell proliferation, synthesis of proteins, and transcription. As predicted, PF-04691502 significantly inhibited tumor growth in P5 PDXs. It also inhibited expression of pAkt, pS6, p4EBP1, and Ki-67, indicating that PF-04691502 probably functions by inhibiting the PI3K/mTOR pathway, consequently suppressing tumor cell proliferation, probably through inhibition of Ki-67 expression. Martin et al. [17] reported that increased Ki-67 staining in prostate cancer is associated with higher PI3K immunohistochemistry scores. In our study, the tumor micromorphology appeared to undergo hyalinization with cell nuclei devitalization after treatment with the PI3K/mTOR inhibitor; this is similar to that seem with radiation, trabectedin, and doxorubicin therapy, as reported by others [18]. This type of change in histologic pattern is probably a common pathological even in MRCL when the therapy is effective. More importantly, side effects were tolerable as weight loss in the mice was never more than 20% with treatment. These results indicate that a PI3K/mTOR inhibitor could have great value for the treatment of MRCL tumors, especially those that are *PIK3CA* mutation positive or those with a high percentage of round cells. Our model could also be used to investigate the interaction between *PIK3CA* and FUS-DDIT3. Recently, de Graaff et al. [19, 20] reported the first spontaneously immortalized myxoid liposarcoma cell line and generated xenograft, which is a big step for MRCL drug research. Unfortunately, this cell line was deteremined to be wildtype for *PIK3CA*, thus could not be used to validate the effect of PI3K/mTOR inhibitors in our study.

Kathleen et al. [21] showed that inhibition of the PI3K pathway could increase tumor lipid content, conferring a more differentiated state, in dedifferentiated liposarcoma xenograft models. Herein, PF-04691502 did not have any observable effect on tumor differentiation as the expression of PPARgamma, ap2, and adipsin was not different between the treatment group and patient tumor sample. This likely indicates that the inhibitory effect of PF-04691502 on MRCL is not due to altering cell differentiation, but is rather based on inhibiting cell proliferation.

In our previous study, we identified many miRNAs that are differentially expressed in each subtype of liposarcoma and normal adipose tissues [22]. Using the model described herein, we also validated the function of these miRNAs and identified novel targets for the treatment of MRCL.

In conclusion, we have successfully established the first PDX model of MRCL, which could be used in future research. We showed that inhibitors targeting the PI3K/mTOR pathway could be effective in treating *PIK3CA* mutation positive MRCL. Our results should be further validated using a larger number of samples.

## MATERIALS AND METHODS

The patient from whom the tumor was derived gave informed consent, and this research was approved by the Ethics Committee of Zhongshan Hospital, Fudan University (Ethical approval number B2012-022).

### PDX establishment

The donor specimen was from a patient known to have MRCL. The patient had multiple surgical excisions and tumor histology signatures were well documented. Several equal-size fragments of tumor tissue from the 4^th^ excision (PA) were carefully harvested from the surgical specimen. These were implanted subcutaneously above the right shoulder blades of 6–8 week-old female BALB/c athymic mice (Shanghai SLAC Laboratory Animal Co., Ltd., Shanghai). When tumor masses grew to 2000 mm^3^ (P0), the P0 tumor was explanted and divided into equal sized specimens and implanted subcutaneously into mice as previously stated. These tumors were defined as P1. In the same manner, P2–P5 generations were established [23]. Tumors were measured using digital calipers (Cal Pro, Sylvac, Switzerland). Tumor volumes were estimated using the formula 0.5 × length × width^2^ (mm^3^).

Tumor fragments of 200 mm^3^ from each passage were submerged into special solution (10% DMSO, 20% FBS, and 70% RPMI 1640 medium) and stored in liquid nitrogen for the establishment of future xenografts. Additional pieces between 500 and 1000 mm^3^ from each passage were either snap-frozen in liquid nitrogen or used for histology.

### Histological analysis

Tumor samples from PDX models (P1, P3, P5) and patient PA were fixed in neutral buffered formalin and embedded in paraffin blocks. Samples were sectioned into 5-μm specimens, mounted on slides, and prepared for histological analysis. Slides were first stained with H&E using a standard protocol.

### FISH

We used the *DDIT3* Break Apart FISH Probe Kit (Abbott Vysis) to test the existence of a *DDIT3* gene fusion. The procedure was performed according to the manufacturer's recommendation.

### Genomic studies

Snap-frozen tumor tissues from each passage were used for genomic analysis. DNA was isolated using a QIAamp DNA mini kit (Qiagen) in accordance with company recommendations. The concentration was quantitated using a NanoDrop ND-1000 spectrophotometer (NanoDrop, Wilmington, DE, USA). DNA samples with A_260/280_ ratios between 1.8 and 2.0 and A_260/230_ ratios above 2.0, and with quality confirmed by gel electrophoresis, were used for WES and SNP 6.0 array analyses.

### Whole exome sequencing

One microgram of each DNA sample, isolated from P5 xenograft tumors, was used for library construction using TruSeq DNA sample preparation kits (Illumina, San Diego, CA, USA). Five hundred nanogram-libraries of DNA from each sample were pooled for exome capturing using the TruSeq Exome Enrichment kit (Illumina). Sequencing was then performed with paired-end 2 × 100 base reads on the Illumina HiSeq 2000 platform (Illumina). Raw. fastq files were first processed by a proprietary algorithm to filter out mouse sequence contamination. We have previously shown that this filter step does not affect human SNP detection [24, 25]. After mouse sequences were removed, data were aligned to the human reference genome hg19/GRCh37 by BWA 0.6.1 and processed using variant calling by GATK 1.6.

### STR analysis

Genomic DNA was extracted from frozen P1 to P5 tumor samples from the PDX model. All samples, together with positive and negative controls, were amplified using the GenePrint 10 System (Promega). Amplified products were then processed using the ABI3730xl Genetic Analyzer. Data were analyzed using GeneMapper4.0 software and then compared to the ATCC, DSMZ, or JCRB databases for reference matching. Ten genetic sites were chosen for detection: CSF1PO, TPOX, TH01, VWA, D5S818, D7S820, D16S539, D13S317, D21S11, and Amelogenin. The similarity rates for each sample were calculated using the equation 2M/N; M was defined as the number of the same repetitive sequence in each site between two groups, and N was the number of all sites chosen for detection (which was 40).

### *PIK3CA* genotyping

DNA was extracted from two 20-μm-thick formalin-fixed paraffin-embedded tissue blocks. One block was from the patient tumor, the other three MRCL tumor samples were chosen as controls. A QIAamp DNA mini kit DNA isolation kit (Qiagen) was used for DNA isolation. Polymerase Chain Reaction (PCR) was used with primers PIK3CA9F 5′- TG CTTTTTCTGTAAATCATCTGTGA-3′, IK3CA9R 5′-CAT GCTGAGATCAGCCAAAT-3′, PIK3CA20F 5′-ATGATGC TTGGCTCTGGAAT-3′, and PIK3CA20R 5′-CCTATG CAATCGGTCTTTGC-3′, with appropriate controls. PCR products were detected by gel electrophoresis using 2% agarose gels and amplified bands were purified using the QIAquick gel extraction kit (Qiagen).

### Compounds and therapeutic assays

After confirming genomic consistency, P5 mice were used to further validate our model. A total of 20 mice were used. Treatment group mice (*n* = 10) were gavaged with the PI3K/mTOR inhibitor PF-04691502 (10 mg/kg) [26, 27] once per day, for 29 days. Control group mice (*n* = 10) were given the same volume of vehicle, once per day, for 29 days. PF-04691502 was suspended in 0.5% methylcellulose. Tumor sizes from both groups were measured twice per week. Tumor volumes were calculated using the modified ellipsoidal formula: V = 0.5 × (length × width^2^) mm^3^ (length = the greatest longitudinal diameter; width = the greatest transverse diameter). All measurements were obtained while mice were conscious. Animals were also weighed twice per week. The mice were euthanized when tumor volumes exceeded 3000 mm^3^ to ensure their wellbeing.

All procedures related to animal handling, care, and treatment were performed according to the guidelines approved by the Institutional Animal Care and Use Committee (IACUC) of WuXi AppTec following guidance from the Association for Assessment and Accreditation of Laboratory Animal Care (AAALAC). The animals were checked daily for any negative effects from tumor growth or treatments, based on normal behavior including mobility, food and water consumption, body weight gain/loss, eye/hair matting, and any other abnormal effects. Death and observed clinical signs were recorded.

### Immunohistochemistry (IHC)

Ten tumor blocks were randomly selected from the two groups (five per group). Tumor slides were immunostained for phosphorylated AKT (pAKT), phosphorylated S6 (pS6), phosphorylated 4EBP1 (p4EBP1), and Ki-67. Briefly, after de-paraffinization and antigen retrieval, the slides were incubated with antibodies against pAKT (1:300, #3787, Cell Signaling), pS6 (1:300, #2215, Cell Signaling), p4EBP1 (1:300, #2855, Cell Signaling), and Ki-67 (1:500, ab66155, Abcam) overnight at 4°C. The slides were then washed and examined using the REAL™ EnVision™ Detection System, Peroxidase/DAB+, Rabbit/Mouse (#K5007, DAKO).

### Western blotting

Western blotting was performed as described previously [28]. Antibodies used were as follows: anti-ap2 (1:50, SC-18661, Santa Cruz Biotechnology), anti-adipsin (1:50, SC-12402, Santa Cruz Biotechnology), and anti-PPARgamma (1:1000, #2435, Cell Signaling).

### Statistical analysis

IHC results were evaluated by two pathologists from Zhongshan Hospital. The data were quantitated according to the range and deepness of color; a score was assigned for each range. A range of 80–100%, was scored as 4; 60–79% was scored as 3, 40–59% was scored as 2, and < 39% was scored as 1. The scores then were multiplied by the depth of color. The depth of the color was divided into three levels, namely, 1, 2, and 3. All analyses were performed using SPSS 16.0 software. The association between experimental and control groups were examined using the independent-samples *t*-test after a normality test; *P* < 0.05 was considered statistically significant. The data depicted are represented as mean ± SD, and graphically represented as mean ± SEM.
